# Oral Microbiome and Alzheimer’s Disease

**DOI:** 10.3390/microorganisms11102550

**Published:** 2023-10-13

**Authors:** Jason Wan, Hongkuan Fan

**Affiliations:** 1 Department of Pathology and Laboratory Medicine, Medical University of South Carolina, Charleston, SC 29425, USA; wanjas1405@gmail.com; 2 Charleston County School of the Arts High School, North Charleston, SC 29405, USA

**Keywords:** oral microbiome, Alzheimer disease, infection

## Abstract

The accumulation of amyloid-beta plaques in the brain is a central pathological feature of Alzheimer’s disease. It is believed that amyloid responses may be a result of the host immune response to pathogens in both the central nervous system and peripheral systems. Oral microbial dysbiosis is a chronic condition affecting more than 50% of older adults. Recent studies have linked oral microbial dysbiosis to a higher brain Aβ load and the development of Alzheimer’s disease in humans. Moreover, the presence of an oral-derived and predominant microbiome has been identified in the brains of patients with Alzheimer’s disease and other neurodegenerative diseases. Therefore, in this opinion article, we aim to provide a summary of studies on oral microbiomes that may contribute to the pathogenesis of the central nervous system in Alzheimer’s disease.

## 1. Introduction

Alzheimer disease (AD) is a neurodegenerative disorder influenced by a complex interplay of environmental epigenetic and genetic factors [1]. AD primarily affects cognition, including behavior, thinking, and memory. The global significance of AD has received increased attention, given that AD and various types of dementia were the 7th leading cause of death worldwide in 2019 [2]. In the US, the prevalence of AD is noteworthy, with approximately 6.7 million Americans aged 65 years and older diagnosed with AD in 2023, a number projected to increase to 13.85 million by 2060, and almost two-thirds are women [3].

The central pathological features of AD involve the accumulation of amyloid-beta (Aβ) plaques and neurofibrillar tangles (NFTs) in the brain. It is believed that amyloid products are a host immune response to pathogens in both the central nervous system (CNS) and peripheral systems. These responses are generated through the protease cleavage of the β-amyloid precursor protein (APP) [4]. APP is a type I transmembrane protein that can be cleaved by various enzymes, including α-Secretase, β-Secretase, and γ-Secretase [4]. Perturbed processing of APP by these enzymes can lead to the production of the key pathogenic amyloid subtype, Aβ42, ultimately leading to the formation of amyloid plaques and the development of AD [4]. Understanding the mechanisms that regulate secretase activity in AD is important for the development of potential therapeutic interventions.

Recent studies reveal a causal link between microbiomes and the pathogenesis of CNS in AD. The microbiome plays a critical role in modulating host immunity and the homeostasis of the oral cavity [5]. All biological and non-biological surfaces in the oral cavity are covered by a microbial biofilm, thus the control of the equilibrium between the host and these microorganisms is vital for the maintenance of both healthy teeth and healthy implants [6]. Gut dysbiosis, which disrupts the balance of bacteria in the gut, is known to impact cognition, likely through the gut–brain axis [7,8,9]. Notably, gut dysbiosis has been observed in patients with AD [10,11,12,13,14,15,16]. Beyond the gut, the oral cavity hosts a diverse community of bacteria that interact with each other and with the host. These oral bacteria can enter into the systemic circulation through activities such as brushing teeth or inflammation of the gingiva, potentially affecting peripheral organs and the CNS [17,18,19,20,21]. While the oral cavity harbors numerous commensal bacteria, it remains unclear whether specific bacteria play a causal role in eliciting pathogenic brain responses. Nevertheless, there is evidence that bacteria found within the human oral cavity can promote Aβ formation and contribute to AD pathology [22,23,24]. In this opinion article, we aim to summarize previous studies that reveal the impact of the oral microbiome on CNS pathogenesis in AD.

## 2. Oral Microbiome and Alzheimer Disease

### 2.1. Porphyromonas gingivalis

*Porphyromonas gingivalis*, commonly referred to as *P. gingivalis*, is a Gram-negative, anaerobic, rod-shaped bacterium. This bacterium can act as an oral pathogen, contributing to the development of chronic periodontitis and potentially serving as a risk factor for the formation of Aβ plaques, cognitive impairment, dementia, and the etiology of AD [23,25,26]. In post-mortem brain samples from AD patients, researchers have detected *P. gingivalis* using qPCR or monoclonal antibodies [27]. Notably, patients with AD exhibited higher blood levels of anti-*P. gingivalis* IgG compared to control subjects, and this increase is associated with specific cognitive impairment [28]. Oral microbiome diversities are lower in patients experiencing cognitive decline when compared to cognitively healthy individuals, suggesting an overgrowth of specific microbiota in patients with cognitive decline [29]. The impact of *P. gingivalis* on CNS pathogenesis related to AD has been revealed through in vitro and in vivo studies using animal models [23,30,31,32,33,34]. In one study, the human neuroblastoma cell line SH-SY5Y produced Aβ40 and Aβ42 in response to *P. gingivalis*, which occurred through the cleavage of the amyloid-β protein precursor in vitro [30]. Moreover, the administration of *P gingivalis* or its lipopolysaccharide (LPS) induced pro-inflammatory responses and Aβ production in the brain, impairing cognitive performance in Sprague Dawley rats, C57BL/6J, and senescence-accelerated mouse prone 8 (SAMP8) mice [31,32]. Sprague Dawley rats are widely used in behavioral and cognitive studies because of their calmness and ease of handling. SAMP8 mice spontaneously overproduce ayloid precursor protein (APP) and exhibit oxidative damage [35]. In male C57BL/6J and SAMP8 mice, intraperitoneally injection of *P. gingivalis* LPS led to a reduction in the expression of neprilysin in the hippocampus [32], and lower levels of neprilysin have been associated with increased Aβ accumulation in AD [36]. In addition, *P. gingivalis* influences the formation of pTau causing the AD pathology [23]. Men exhibit a higher abundance of the genus *Porphyromonas* in the saliva compared to women, but no data have shown sex differences in oral *P. gingivalis* enrichment [37].

### 2.2. Actinobacillus actinomycetemcomitans

The bacterium *A. actinomycetemcomitans*, also called *Aggregatibacter*, is a Gram-negative, facultative anaerobic bacillus known to be a causative agent of periodontal diseases. The main pathogenic factors attributed to *A. actinomycetemcomitans* include leukotoxin, LPS, surface-associated materials, and various enzymes [38,39]. This bacterium is further classified into five serotypes (a-e) [38]. Particularly, LPS from serotype B has been shown to trigger high levels of proinflammatory cytokine production in microglia and an increase the secretion of the Aβ1-42 peptide in mixed hippocampal cells in vitro. These findings are significant as they are linked to the pathogenesis of AD [40]. No data have shown sex differences between oral differences in either that genus of bacteria or *A. actinomycetemcomitans* [37].

### 2.3. Tannerella forsythia and Treponema denticola

*Tannerella forsythia*, along with *P. gingivalis*, and *Treponema denticola* constitute the three major oral microbiomes associated with the etiology of chronic periodontitis and are more prevalent in patients suffering from this condition [41,42]. *T. forsythia* is a Gram-negative anaerobic bacterium that primarily resides in the subgingival cavity and is known to promote bone loss, a factor implicated in the development of periodontitis. Previous studies have unveiled that *T. forsythia* induces periodontal bone loss in mice, a process dependent on bacterial BspA protein and the host’s TLR2 receptors using TLR2−/− mice [43]. Additionally, Th2 cells have been shown to play pathogenic roles in *T. forsythia*-induced bone loss using STAT6−/− and TLR2−/− mice on BALB/cJ backgrounds [44]. Moreover, atherosclerosis-prone apolipoprotein E-deficient (Apoe−/−) mice display a marked increase in total plasma cholesterol levels. Moreover, when *T. forsythia* is orally inoculated in ApoE^null^ mice, it results in increased serum amyloid A, decreased serum nitric oxide, and elevated serum lipoproteins compared to control groups. These findings suggest a potential role of oral *T. forsythia* in accelerating atherosclerosis in hyperlipidemic mice [45], but an association with the development of AD is lacking.

*T. denticola*, along with *P. gingivalis*, and *T. forsythia* constitute the “red complex,” which is a recognized polybacterial pathogenic consortium in periodontitis. These bacteria are key components of a local dysbiosis in the oral microbiome and contribute to alterations in the host’s immune response [46]. Notably, *Treponema* species have been identified in the brains of patients with AD [27]. In animal studies, oral inoculation of T. denticola into C57BL/6 mice has been shown to result in the production of Aβ1–42 in the hippocampus and neuronal apoptosis, suggesting a potential link to memory impairment [47,48].

In summary, there is evidence to suggest that *T. denticola* may have the potential to cause neurodegeneration through the periodontal route of infection. No data have shown sex differences between oral differences in either that genus of bacteria or *T. forsythia* or *T. denticola* [37]. Moreover, the evidence supporting *T. forsythia* as a periodontal pathogen includes its prevalence in patients with periodontitis, host responses to infection, the induction of diseases in animal models upon oral inoculation, and the potential impact of its virulence factors on disease pathogenesis and progression [44,45,49,50]. However, it is important to emphasize that although patients with periodontal diseases are at an elevated risk of developing AD, the direct evidence implicating *T. forsythia* as a causative agent of AD remains lacking and requires further investigations.

### 2.4. Streptococcus mutans

*Streptococcus* bacteria have been detected in the brain microbiome from AD patients [51]. *S. mutans* is a Gram-positive bacterium with acidogenicity and contributes to the etiology of dental caries development [52]. Women exhibit a higher abundance of other species of *Streptococcus* bacteria (e.g., *S. parasanguinis*) in the saliva compared to men, but no data have shown sex differences in oral *S. mutans* enrichment [37]. Previous research has provided evidence that *S. mutans* is capable of producing amyloid, as demonstrated in both laboratory and clinically isolated strains. Amyloid has also been detected in human dental plaque. These findings strongly suggest that *S. mutans* can function as an amyloid-forming organism, capable of forming recalcitrant biofilms and cavities, and generate acids from dietary sugars [22]. Furthermore, specific antigens derived from *S. mutans* walls, such as amyloidogenic adhesin P1 and WapA, can also form amyloid fibrils and influence biofilm development, indicating that these specific bacterial cell wall proteins can be therapeutic targets of anti-amyloid compounds [53].

Interestingly, about 20% clinical isolates of *S. mutans* have the *cnm* gene, which codes for a glycosylated collagen- and laminin-binding surface adhesion. This adhesion is associated with infection and an elevated risk of caries development. Computer analysis has predicted that cnm may play a role in amyloid aggregation through the collagen-binding domain (CBD) of cnm. Moreover, cnm has been identified as a major amyloidogenic protein in *S. mutans* biofilms. When cnm is in a monomer state, it promotes attachment to collagenous substrates through its CBD. However, when it aggregates, it loses its collagen-binding ability, likely contributing to the formation of the biofilm matrix. These findings highlight the connection between functional amyloids and the pathobiology and ecology of *S. mutans* [54]. Nevertheless, it is important to note that direct evidence of a causal link between *S. mutans*-mediated amyloid production in the brain in vivo and AD is currently lacking.

### 2.5. Fusobacterium nucleatum

*F. nucleatum* is a Gram-negative anaerobic bacterium typically found in the oral commensal microbiome, occasionally displaying opportunistic pathogenic behavior [55]. Men exhibit a higher abundance of the genus *Fusobacterium* in the saliva compared to women, but no data have shown sex differences in oral *F. nulceatum* enrichment [37]. *F. nucleatum* has been associated with various human diseases, including periodontal diseases, adverse pregnancy outcome, and oral and colon cancers [56,57,58]. Recent studies suggest a potential link between *F. nucleatum* and the development and pathogenesis of AD. One study reported the production of an adhesion called FadA by *F. nucleatum* under stressful and unhealthy conditions. FadA exhibits amyloid-like properties through a Fap2-like autotransporter mechanism. This amyloid-like FadA may serve as a scaffold for biofilm formation and displays resistance to acidic environments. While these findings shed light on how *F. nucleatum* contributes to the formation of a stable amyloid-like structure, the precise mechanisms and causal relationships are still under investigation and are not fully understood. Nonetheless, *F. nucleatum* infection has been linked to increased systemic or local inflammation, which ultimately impacts blood–brain barrier permeability. This, in turn, leads to systemic and CNS immune activation, CNS-related neuroinflammation, the accumulation of Aβ plaques in the brain, and the progression of AD.

### 2.6. Actinomycin meyeri

*A. meyeri* is a Gram-positive facultative anaerobic bacterium. Previous clinical reports have indicated that oral infections caused by *A. meyeri* are associated with brain infections or dysfunction [59]. Elevated levels of oral *Actinomyces* have been observed in patients with chronic periodontitis, strongly suggesting a link between chronic periodontitis and an increased risk of cognitive decline and AD [60]. Notably, *Actinomycetia* [61], a class-level bacteria of *A. meyeri*, and *Actinomyces* [62], a genus-level bacteria of *A. meyeri*, have been found to be increased in the brain of AD patients compared to the non-AD controls. In support of this finding, *Actinobacteria*, also known as *Actinomycetes* [63], are commonly found in the oral cavity and are dysbiotic gastrointestinal microbiota [64,65]. Importantly, oral inoculation of *A. meyeri*, as opposed to *A. odontolyticus* and *Neisseria elongate*, led to proinflammatory myeloid cell infiltration in the brain (associated with long-term memory decline [66]) and a robust increase in cortical Aβ plaques (averaging a 120% increase) in C57/B6 mice when compared to a control group receiving saline [24]. However, further investigations are needed to elucidate the mechanisms responsible for *A. meyeri*-mediated Aβ plaques formation in the CNS, along with additional evidence in AD patients. Women exhibit a higher abundance of other species of *Actinomycin* bacteria (e.g., *A. viscosus*) in the saliva compared to men, but no data have shown sex differences in oral *A. meyeri* enrichment [37].

## 3. Discussion

Several risk factors contribute to the development of AD, including lifestyle choices, infections or inflammation, as well as genetic and environmental factors [67]. Among these, chronic oral inflammatory conditions, such as periodontitis, hold a distinctive position due to their anatomical proximity to the brain [60]. Periodontitis is a microbiome-related chronic inflammatory disease affecting individuals of all age groups and often becomes chronic among the older population [60]. Of the three known periodontal microbiomes, *P. gingivalis*, *T. forsythia*, and *T. denticola*, it has been reported that they play a role in promoting inflammatory pathologies in the CNS associated with AD [60]. However, other oral pathogens may also contribute to local and CNS inflammation and the development of AD. These oral pathogenic microbiomes, through everyday activities such as brushing teeth, flossing, and chewing food, not only serve as sources of chronic infection and inflammation but also disperse their products into the brain through the oral–brain axis pathways [18]. The long-term effects of microbial products, virulence factors, and inflammatory mediators on CNS pathogenesis may involve their entry into the brain, thereby continuously stimulating CNS immune cells like microglia. This, in turn, triggers an immune response, regulating neuroinflammation, amyloid beta production, and neuronal cell apoptosis, which are hallmark features of AD pathogenesis. These immune disturbances within the CNS can subsequently cause functional impairments, such as memory deficits. In this review, we discuss the potential mechanisms through which specific oral pathogenic microbiomes may contribute to the AD development.

Oral bacteria have been observed to translocate into the blood stream, even in healthy individuals, following routine activities like tooth brushing [17,18,19]. Some of these translocated bacteria have been implicated in contributing to the development of various diseases [68,69,70]. In fact, non-protein components of bacterial cell walls have been detected in human plasma samples under physiological conditions, even in the absence of clinical infections [71]. These components, such as bacterial peptidoglycan and LPS, have also been identified in the brain tissues of patients with AD [61,72]. Further, exposure to viral or bacterial pathogens has been shown to upregulate the expression of neuronal Aβ in non-transformed cell culture models and in the brains of wild-type rats. This phenomenon may represent an antimicrobial defense response [73]. Intriguingly, the depletion of CD14+ cells improves AD neuropathogenesis [74]. Thus, microbial pathogens and the associated antimicrobial responses may play a pivotal role in the etiology and pathogenesis of AD.

**Figure 1:**
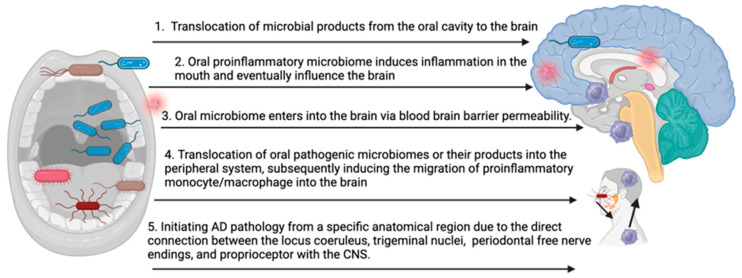
Potential mechanisms of oral microbiome-induced brain pathology in AD (bioRender).

The precise mechanisms underlying the impact of the oral pathogenic microbiome on the development and progression of AD remain incompletely understood. The accumulation of key pathogenic factors, such as amyloid beta and Tau, in the brain may result from several possibilities (Figure 1): (1) translocation of microbial products from the oral cavity to the brain, leading to repeated stimulation of CNS immune cells, (2) oral inflammation influencing CNS inflammation, (3) the oral microbiome enters into the brain via blood–brain barrier permeability, (4) translocation of oral pathogenic microbiomes or their products into the peripheral system, subsequently inducing the migration of proinflammatory monocyte/macrophage into the brain, and (5) initiating AD pathology from a specific anatomical region due to the direct connection between the locus coeruleus, trigeminal nuclei, periodontal free nerve endings, and proprioceptors with the CNS. Each of these pathways represents a potential route through which oral pathogenic microbiomes may contribute to the onset and progression of AD.

In summary, AD is a complex condition influenced by multiple potential contributing factors. Recent research reveals that the deposition of amyloid in the brain may be an immune response to pathogens or foreign antigens. While extensive studies have focused on the gut microbiome and its relationship to neuropathogenesis, there has been limited investigation into the connection between the oral microbiome and AD. In this review, we explore a unique association between oral microbial dysbiosis and the neuropathogenesis in AD. If a definitive and causative link between the oral microbiome and AD development or progression is proven, assessing specific microbiome abundance in the oral cavity may serve as a valuable risk biomarker for the early stage of AD or as a predictor of AD progression. Rational interventions to modify the oral microbiota could potentially offer a promising strategy to delay the onset of AD or prevent its progression.

## References

[1] Armstrong M.J., Jin Y., Vattathil S.M., Huang Y., Schroeder J.P., Bennet D.A., Qin Z.S., Wingo T.S., Jin P. (2023). Role of TET1-mediated epigenetic modulation in Alzheimer’s disease. Neurobiol. Dis..

[2] Abbayya K., Puthanakar N.Y., Naduwinmani S., Chidambar Y.S. (2015). Association between Periodontitis and Alzheimer’s Disease. N. Am. J. Med. Sci..

[3] Rajan K.B., Weuve J., Barnes L.L., McAninch E.A., Wilson R.S., Evans D.A. (2021). Population estimate of people with clinical Alzheimer’s disease and mild cognitive impairment in the United States (2020–2060). Alzheimers Dement..

[4] Jungbauer G., Stahli A., Zhu X., Auber Alberi L., Sculean A., Eick S. (2022). Periodontal microorganisms and Alzheimer disease—A causative relationship?. Periodontol. 2000.

[5] Narayan N.R., Mendez-Lagares G., Ardeshir A., Lu D., Van Rompay K.K., Hartigan-O’Connor D.J. (2015). Persistent effects of early infant diet and associated microbiota on the juvenile immune system. Gut Microbes.

[6] Bianchi S., Fantozzi G., Bernardi S., Antonouli S., Continenza M.A., Macchiarelli G. (2020). Commercial oral hygiene products and implant collar surfaces: Scanning electron microscopy observations. Can. J. Dent. Hyg..

[7] Stilling R.M., Dinan T.G., Cryan J.F. (2014). Microbial genes, brain & behaviour—Epigenetic regulation of the gut-brain axis. Genes Brain Behav..

[8] Stilling R.M., van de Wouw M., Clarke G., Stanton C., Dinan T.G., Cryan J.F. (2016). The neuropharmacology of butyrate: The bread and butter of the microbiota-gut-brain axis?. Neurochem. Int..

[9] Panee J., Gerschenson M., Chang L. (2018). Associations between Microbiota, Mitochondrial Function, and Cognition in Chronic Marijuana Users. J. Neuroimmune Pharmacol..

[10] Liu M., Zhong P. (2022). Modulating the Gut Microbiota as a Therapeutic Intervention for Alzheimer’s Disease. Indian J. Microbiol..

[11] Benichou Haziot C., Birak K.S. (2023). Therapeutic Potential of Microbiota Modulation in Alzheimer’s Disease: A Review of Preclinical Studies. J. Alzheimers Dis. Rep..

[12] Cammann D., Lu Y., Cummings M.J., Zhang M.L., Cue J.M., Do J., Ebersole J., Chen X., Oh E.C., Cummings J.L. (2023). Genetic correlations between Alzheimer’s disease and gut microbiome genera. Sci. Rep..

[13] Das T.K., Ganesh B.P. (2023). Interlink between the gut microbiota and inflammation in the context of oxidative stress in Alzheimer’s disease progression. Gut Microbes.

[14] Jemimah S., Chabib C.M.M., Hadjileontiadis L., AlShehhi A. (2023). Gut microbiome dysbiosis in Alzheimer’s disease and mild cognitive impairment: A systematic review and meta-analysis. PLoS ONE.

[15] Susmitha G., Kumar R. (2023). Role of microbial dysbiosis in the pathogenesis of Alzheimer’s disease. Neuropharmacology.

[16] Thakkar A., Vora A., Kaur G., Akhtar J. (2023). Dysbiosis and Alzheimer’s disease: Role of probiotics, prebiotics and synbiotics. Naunyn Schmiedebergs Arch Pharmacol..

[17] Bahrani-Mougeot F.K., Paster B.J., Coleman S., Ashar J., Barbuto S., Lockhart P.B. (2008). Diverse and novel oral bacterial species in blood following dental procedures. J. Clin. Microbiol..

[18] Lockhart P.B., Brennan M.T., Sasser H.C., Fox P.C., Paster B.J., Bahrani-Mougeot F.K. (2008). Bacteremia associated with toothbrushing and dental extraction. Circulation.

[19] Kaplan E.L. (2009). Letter by Kaplan regarding article, “Bacteremia associated with toothbrushing and dental extraction”. Circulation.

[20] Han Y.W., Wang X. (2013). Mobile microbiome: Oral bacteria in extra-oral infections and inflammation. J. Dent. Res..

[21] Tsukasaki M., Komatsu N., Nagashima K., Nitta T., Pluemsakunthai W., Shukunami C., Iwakura Y., Nakashima T., Okamoto K., Takayanagi H. (2018). Host defense against oral microbiota by bone-damaging T cells. Nat. Commun..

[22] Oli M.W., Otoo H.N., Crowley P.J., Heim K.P., Nascimento M.M., Ramsook C.B., Lipke P.N., Brady L.J. (2012). Functional amyloid formation by Streptococcus mutans. Microbiology.

[23] Dominy S.S., Lynch C., Ermini F., Benedyk M., Marczyk A., Konradi A., Nguyen M., Haditsch U., Raha D., Griffin C. (2019). Porphyromonas gingivalis in Alzheimer’s disease brains: Evidence for disease causation and treatment with small-molecule inhibitors. Sci. Adv..

[24] Luo Z., Fitting S., Robinson C., Benitez A., Li M., Wu Y., Fu X., Amato D., Ning W., Funderburg N. (2021). Chronic cannabis smoking-enriched oral pathobiont drives behavioral changes, macrophage infiltration, and increases beta-amyloid protein production in the brain. EBioMedicine.

[25] Wu Z., Ni J., Liu Y., Teeling J.L., Takayama F., Collcutt A., Ibbett P., Nakanishi H. (2017). Cathepsin B plays a critical role in inducing Alzheimer’s disease-like phenotypes following chronic systemic exposure to lipopolysaccharide from Porphyromonas gingivalis in mice. Brain Behav. Immun..

[26] Zhan X., Stamova B., Sharp F.R. (2018). Lipopolysaccharide Associates with Amyloid Plaques, Neurons and Oligodendrocytes in Alzheimer’s Disease Brain: A Review. Front. Aging Neurosci..

[27] Riviere G.R., Riviere K.H., Smith K.S. (2002). Molecular and immunological evidence of oral Treponema in the human brain and their association with Alzheimer’s disease. Oral Microbiol. Immunol..

[28] Noble J.M., Borrell L.N., Papapanou P.N., Elkind M.S., Scarmeas N., Wright C.B. (2009). Periodontitis is associated with cognitive impairment among older adults: Analysis of NHANES-III. J. Neurol. Neurosurg. Psychiatry.

[29] Da D., Zhao Q., Zhang H., Wu W., Zeng X., Liang X., Jiang Y., Xiao Z., Yu J., Ding S. (2023). Oral microbiome in older adults with mild cognitive impairment. J. Oral Microbiol..

[30] Kanagasingam S., von Ruhland C., Welbury R., Chukkapalli S.S., Singhrao S.K. (2022). Porphyromonas gingivalis Conditioned Medium Induces Amyloidogenic Processing of the Amyloid-beta Protein Precursor upon in vitro Infection of SH-SY5Y Cells. J. Alzheimers Dis. Rep..

[31] Jin R., Ning X., Liu X., Zhao Y., Ye G. (2023). Porphyromonas gingivalis-induced periodontitis could contribute to cognitive impairment in Sprague-Dawley rats via the P38 MAPK signaling pathway. Front. Cell Neurosci..

[32] Morikawa T., Uehara O., Paudel D., Yoshida K., Harada F., Hiraki D., Sato J., Matsuoka H., Kuramitsu Y., Michikawa M. (2023). Systemic Administration of Lipopolysaccharide from Porphyromonas gingivalis Decreases Neprilysin Expression in the Mouse Hippocampus. In Vivo.

[33] Wang L., Liang D., Huang Y., Chen Y., Yang X., Huang Z., Jiang Y., Su H., Wang L., Pathak J.L. (2023). SAP deficiency aggravates periodontitis possibly via C5a-C5aR signaling-mediated defective macrophage phagocytosis of Porphyromonas gingivalis. J. Adv. Res..

[34] Zhang Y., Sun Y., Hu Y., Zheng S., Shao H., Lin L., Pan Y., Li C. (2023). Porphyromonas gingivalis msRNA P.G_45033 induces amyloid-beta production by enhancing glycolysis and histone lactylation in macrophages. Int. Immunopharmacol..

[35] Morley J.E., Armbrecht H.J., Farr S.A., Kumar V.B. (2012). The senescence accelerated mouse (SAMP8) as a model for oxidative stress and Alzheimer’s disease. Biochim. Biophys. Acta.

[36] Carpentier M., Robitaille Y., DesGroseillers L., Boileau G., Marcinkiewicz M. (2002). Declining expression of neprilysin in Alzheimer disease vasculature: Possible involvement in cerebral amyloid angiopathy. J. Neuropathol. Exp. Neurol..

[37] Liu X., Tong X., Jie Z., Zhu J., Tian L., Sun Q., Ju Y., Zou L., Lu H., Qiu X. (2023). Sex differences in the oral microbiome, host traits, and their causal relationships. iScience.

[38] Gholizadeh P., Pormohammad A., Eslami H., Shokouhi B., Fakhrzadeh V., Kafil H.S. (2017). Oral pathogenesis of Aggregatibacter actinomycetemcomitans. Microb. Pathog..

[39] Nagasawa T., Kato S., Furuichi Y. (2021). Evaluation of the Virulence of Aggregatibacter actinomycetemcomitans Through the Analysis of Leukotoxin. Methods Mol. Biol..

[40] Diaz-Zuniga J., Munoz Y., Melgar-Rodriguez S., More J., Bruna B., Lobos P., Monasterio G., Vernal R., Paula-Lima A. (2019). Serotype b of Aggregatibacter actinomycetemcomitans triggers pro-inflammatory responses and amyloid beta secretion in hippocampal cells: A novel link between periodontitis and Alzheimer’s disease?. J. Oral Microbiol..

[41] Haigh R.D., Crawford L.A., Ralph J.D., Wanford J.J., Vartoukian S.R., Hijazi K., Wade W., Oggioni M.R. (2017). Draft Whole-Genome Sequences of Periodontal Pathobionts Porphyromonas gingivalis, Prevotella intermedia, and Tannerella forsythia Contain Phase-Variable Restriction-Modification Systems. Genome Announc..

[42] Tomita S., Komiya-Ito A., Imamura K., Kita D., Ota K., Takayama S., Makino-Oi A., Kinumatsu T., Ota M., Saito A. (2013). Prevalence of Aggregatibacter actinomycetemcomitans, Porphyromonas gingivalis and Tannerella forsythia in Japanese patients with generalized chronic and aggressive periodontitis. Microb. Pathog..

[43] Sharma A., Inagaki S., Honma K., Sfintescu C., Baker P.J., Evans R.T. (2005). Tannerella forsythia-induced alveolar bone loss in mice involves leucine-rich-repeat BspA protein. J. Dent. Res..

[44] Myneni S.R., Settem R.P., Connell T.D., Keegan A.D., Gaffen S.L., Sharma A. (2011). TLR2 signaling and Th2 responses drive Tannerella forsythia-induced periodontal bone loss. J. Immunol..

[45] Chukkapalli S.S., Rivera-Kweh M.F., Velsko I.M., Chen H., Zheng D., Bhattacharyya I., Gangula P.R., Lucas A.R., Kesavalu L. (2015). Chronic oral infection with major periodontal bacteria Tannerella forsythia modulates systemic atherosclerosis risk factors and inflammatory markers. Pathog. Dis..

[46] Holt S.C., Ebersole J.L. (2005). Porphyromonas gingivalis, Treponema denticola, and Tannerella forsythia: The “red complex”, a prototype polybacterial pathogenic consortium in periodontitis. Periodontol. 2000.

[47] Su X., Tang Z., Lu Z., Liu Y., He W., Jiang J., Zhang Y., Wu H. (2021). Oral Treponema denticola Infection Induces Abeta(1-40) and Abeta(1-42) Accumulation in the Hippocampus of C57BL/6 Mice. J. Mol. Neurosci..

[48] Wu L., Su X., Tang Z., Jian L., Zhu H., Cheng X., Wu H. (2022). Treponema denticola Induces Neuronal Apoptosis by Promoting Amyloid-beta Accumulation in Mice. Pathogens.

[49] Socransky S.S. (1979). Criteria for the infectious agents in dental caries and periodontal disease. J. Clin. Periodontol..

[50] Kesavalu L., Sathishkumar S., Bakthavatchalu V., Matthews C., Dawson D., Steffen M., Ebersole J.L. (2007). Rat model of polymicrobial infection, immunity, and alveolar bone resorption in periodontal disease. Infect. Immun..

[51] Hu Z., McKenzie C.A., Smith C., Haas J.G., Lathe R. (2023). The remarkable complexity of the brain microbiome in health and disease. BioRxiv.

[52] Guo L., Hu W., He X., Lux R., McLean J., Shi W. (2013). investigating acid production by Streptococcus mutans with a surface-displayed pH-sensitive green fluorescent protein. PLoS ONE.

[53] Besingi R.N., Wenderska I.B., Senadheera D.B., Cvitkovitch D.G., Long J.R., Wen Z.T., Brady L.J. (2017). Functional amyloids in Streptococcus mutans, their use as targets of biofilm inhibition and initial characterization of SMU_63c. Microbiology.

[54] di Cologna N.M., Samaddar S., Valle C.A., Vargas J., Aviles-Reyes A., Morales J., Ganguly T., Pileggi R., Brady L.J., Lemos J.A. (2021). Amyloid Aggregation of Streptococcus mutans Cnm Influences Its Collagen-Binding Activity. Appl. Environ. Microbiol..

[55] Meng Q., Gao Q., Mehrazarin S., Tangwanichgapong K., Wang Y., Huang Y., Pan Y., Robinson S., Liu Z., Zangiabadi A. (2021). Fusobacterium nucleatum secretes amyloid-like FadA to enhance pathogenicity. EMBO Rep..

[56] Figuero E., Han Y.W., Furuichi Y. (2020). Periodontal diseases and adverse pregnancy outcomes: Mechanisms. Periodontol. 2000.

[57] Rubinstein M.R., Baik J.E., Lagana S.M., Han R.P., Raab W.J., Sahoo D., Dalerba P., Wang T.C., Han Y.W. (2019). Fusobacterium nucleatum promotes colorectal cancer by inducing Wnt/beta-catenin modulator Annexin A1. EMBO Rep..

[58] Han Y.W. (2015). Fusobacterium nucleatum: A commensal-turned pathogen. Curr. Opin. Microbiol..

[59] Clancy U., Ronayne A., Prentice M.B., Jackson A. (2015). Actinomyces meyeri brain abscess following dental extraction. BMJ Case Rep..

[60] Teixeira F.B., Saito M.T., Matheus F.C., Prediger R.D., Yamada E.S., Maia C.S.F., Lima R.R. (2017). Periodontitis and Alzheimer’s Disease: A Possible Comorbidity between Oral Chronic Inflammatory Condition and Neuroinflammation. Front. Aging Neurosci..

[61] Siddiqui H., Eribe Ribs E.K., Singhrao S.K., Olsen I. (2019). High Throughput Sequencing Detects Gingivitis and Periodontal Oral Bacteria in Alzheimer’s Disease Autopsy Brains. J. Neurosci. Res..

[62] Fu K.L., Chiu M.J., Wara-Aswapati N., Yang C.N., Chang L.C., Guo Y.L., Ni Y.H., Chen Y.W. (2022). Oral microbiome and serological analyses on association of Alzheimer’s disease and periodontitis. Oral Dis..

[63] Nouioui I., Carro L., Garcia-Lopez M., Meier-Kolthoff J.P., Woyke T., Kyrpides N.C., Pukall R., Klenk H.P., Goodfellow M., Goker M. (2018). Genome-Based Taxonomic Classification of the Phylum Actinobacteria. Front. Microbiol..

[64] Zhuang Z.Q., Shen L.L., Li W.W., Fu X., Zeng F., Gui L., Lu Y., Cai M., Zhu C., Tan Y.L. (2018). Gut Microbiota is Altered in Patients with Alzheimer’s Disease. J. Alzheimers Dis..

[65] Alonso R., Pisa D., Fernandez-Fernandez A.M., Carrasco L. (2018). Infection of Fungi and Bacteria in Brain Tissue From Elderly Persons and Patients With Alzheimer’s Disease. Front. Aging Neurosci..

[66] Yamasaki R., Lu H., Butovsky O., Ohno N., Rietsch A.M., Cialic R., Wu P.M., Doykan C.E., Lin J., Cotleur A.C. (2014). Differential roles of microglia and monocytes in the inflamed central nervous system. J. Exp. Med..

[67] Shoemark D.K., Allen S.J. (2015). The microbiome and disease: Reviewing the links between the oral microbiome, aging, and Alzheimer’s disease. J. Alzheimers Dis..

[68] Durand R., Gunselman E.L., Hodges J.S., Diangelis A.J., Michalowicz B.S. (2009). A pilot study of the association between cariogenic oral bacteria and preterm birth. Oral Dis..

[69] Patrakka O., Pienimaki J.P., Tuomisto S., Ollikainen J., Lehtimaki T., Karhunen P.J., Martiskainen M. (2019). Oral Bacterial Signatures in Cerebral Thrombi of Patients With Acute Ischemic Stroke Treated With Thrombectomy. J. Am. Heart Assoc..

[70] Dasanayake A.P., Li Y., Wiener H., Ruby J.D., Lee M.J. (2005). Salivary Actinomyces naeslundii genospecies 2 and Lactobacillus casei levels predict pregnancy outcomes. J. Periodontol..

[71] Azzouz D., Omarbekova A., Heguy A., Schwudke D., Gisch N., Rovin B.H., Caricchio R., Buyon J.P., Alekseyenko A.V., Silverman G.J. (2019). Lupus nephritis is linked to disease-activity associated expansions and immunity to a gut commensal. Ann. Rheum. Dis..

[72] Poole S., Singhrao S.K., Kesavalu L., Curtis M.A., Crean S. (2013). Determining the presence of periodontopathic virulence factors in short-term postmortem Alzheimer’s disease brain tissue. J. Alzheimers Dis..

[73] Kumar D.K.V., Choi S.H., Washicosky K.J., Eimer W.A., Tucker S., Ghofrani J., Lefkowitz A., McColl G., Goldstein L.E., Tanzi R.E. (2016). Amyloid-β peptide protects against microbial infection in mouse and worm models of Alzheimer’s disease. Sci. Transl. Med..

[74] Reed-Geaghan E.G., Reed Q.W., Cramer P.E., Landreth G.E. (2010). Deletion of CD14 attenuates Alzheimer’s disease pathology by influencing the brain’s inflammatory milieu. J. Neurosci..

